# P-38. Impact of Rapid Diagnostic Testing of Blood Cultures on Time to Optimal Therapy at an Academic Medical Center Versus Community Hospitals

**DOI:** 10.1093/ofid/ofaf695.267

**Published:** 2026-01-11

**Authors:** Lauren Lee, Noor Zaidan, Rachel S Britt, Natalie Williams-Bouyer

**Affiliations:** Memorial Hermann Katy Hospital, Houston, TX; UTMB, Galveston, Texas; UTMB Health, Galveston, Texas; University of Texas Medical Branch, Galveston, Texas

## Abstract

**Background:**

Rapid diagnostic tests (RDTs) can minimize the time from blood culture collection to organism identification, preventing delays in the start of optimal antimicrobial therapy. Verigene®, a RDT, is available at the University of Texas Medical Branch (UTMB Health) academic medical center (AMC) in Galveston, Texas. UTMB Health's community hospitals do not have RDT technology locally. The purpose of this study was to compare the impact of RDTs on the time to optimal therapy (TTOT) at an AMC versus community hospitals.

**Methods:**

This retrospective chart review study between January to June 2023 included patients ≥ 18 years old with positive blood cultures for *Staphylococcus aureus*. Patients who were not admitted, pregnant, incarcerated, had polymicrobial blood cultures, or on appropriate therapy before RDT results, were excluded. Baseline characteristics included age, sex, Charlson Comorbidity Index (CCI), and an infectious diseases (ID) consult placement. Categorical and continuous data were analyzed using Chi-squared/Fisher’s exact and Student’s t-test, respectively. A p-value of < 0.05 was statistically significant. The primary outcome was TTOT (in hours) from blood culture collection. Optimal therapy was defined as de-escalation or escalation of antimicrobials based on the *mecA gene* detection. Secondary outcomes included time from blood culture collection to time of Gram stain and RDT results, length of stay, and 30-day all-cause mortality rate.

**Results:**

Out of the 84 patient charts reviewed, 53 met inclusion criteria. Baseline characteristics were similar across all sites. The TTOT was shorter at the AMC by a median of 10.83 hours (32.09 hours vs 42.92 hours, p=0.03). The time from blood culture collection to Gram stain were similar at all sites (16.49 hours vs 18.57 hours, p=0.29). The AMC had a shorter time from blood culture collection to RDT results by a median 6.51 hours (18.65 hours vs 25.16 hours, p< 0.001). The length of stay was 4 days longer at the AMC (14 vs 10 days, p=0.04). The 30-day all-cause mortality rate was similar across all sites (18.18% vs 22.58%, p=0.75).

**Conclusion:**

The on-site RDTs significantly decreased the TTOT and the time from blood culture collection to organism identification. The results of the study will assist the implementation of RDTs at all hospitals.

**Disclosures:**

All Authors: No reported disclosures

